# Kondo-like Behavior in Lightly Gd-Doped Manganite CaMnO_3_

**DOI:** 10.3390/nano15110784

**Published:** 2025-05-23

**Authors:** Tomislav Ivek, Matija Čulo, Nikolina Novosel, Maria Čebela, Bojana Laban, Uroš Čakar, Milena Rosić

**Affiliations:** 1 Institut za fiziku, Bijenička cesta 46, HR-10000 Zagreb, Croatiannovosel@ifs.hr (N.N.); 2 “Vinča” Institute of Nuclear Sciences, National Institute of the Republic of Serbia, University of Belgrade, Mike Petrovića Alasa 12-14, 11351 Belgrade, Serbia; mcebela@vin.bg.ac.rs (M.Č.); mrosic@vin.bg.ac.rs (M.R.); 3 Faculty of Sciences and Mathematics, University of Priština in Kosovska Mitrovica, Lole Ribara 29, 38220 Kosovska Mitrovica, Serbia; bojana.laban@pr.ac.rs; 4 Department of Bromatology, Faculty of Pharmacy, University of Belgrade, 11000 Belgrade, Serbia; urosc@pharmacy.bg.ac.rs

**Keywords:** manganites, magnetotransport, resistivity upturn, Kondo-like scattering, percolation

## Abstract

Manganese oxides (manganites) are among the most studied materials in condensed matter physics due to the famous colossal magnetoresistance and very rich phase diagrams characterized by strong competition between ferromagnetic (FM) metallic and antiferromagnetic (AFM) insulating phases. One of the key questions that remains open even after more than thirty years of intensive research is the exact conductivity mechanism in insulating as well as in metallic phases and its relation to the corresponding magnetic structure. In order to shed more light on this problem, here, we report magnetotransport measurements on sintered nanocrystalline samples of the very poorly explored manganites ${\mathrm{Ca}}_{1-x}{\mathrm{Gd}}_{x}{\mathrm{MnO}}_{3}$ with x=0.05 and x=0.10, in the temperature range 2–300 K, and in magnetic fields up to 16 T. Our results indicate that both compounds at low temperatures exhibit metallic behavior with a peculiar resistivity upturn and a large negative magnetoresistance. We argue that such behavior is consistent with a Kondo-like scattering on Gd impurities coupled with the percolation of FM metallic regions within insulating AFM matrix.

## 1. Introduction

In contrast to conventional materials, in many advanced materials there is strong coupling between charge, spin, lattice, and orbital degrees of freedom, which highly complicates the understanding of these materials but also leads to many exotic and applicable phenomena. One of the most prominent examples of such material is the family of manganese oxides (manganites) described by a general chemical formula $R_{1-x}A_{x}{\mathrm{MnO}}_{3}$, where *R* is a rare-earth element or Bi and *A* is an alkaline-earth element or Pb. Significant large interest in manganites started in 1994 with the discovery of the famous colossal magnetoresistance (CMR), an enormous decrease in electrical resistivity (ρ) in an applied magnetic field (*H*), which close to the Curie temperature can reach values as high as 127,000 % [1], in contrast to the conventional materials, where the effect of *H* is usually a few %. Aside from CMR, manganites are also interesting because of their very rich phase diagrams in which the ferromagnetic (FM) phase is usually metallic, while the antiferromagnetic (AFM) phase is insulating [2,3,4,5].

**Figure 1:**
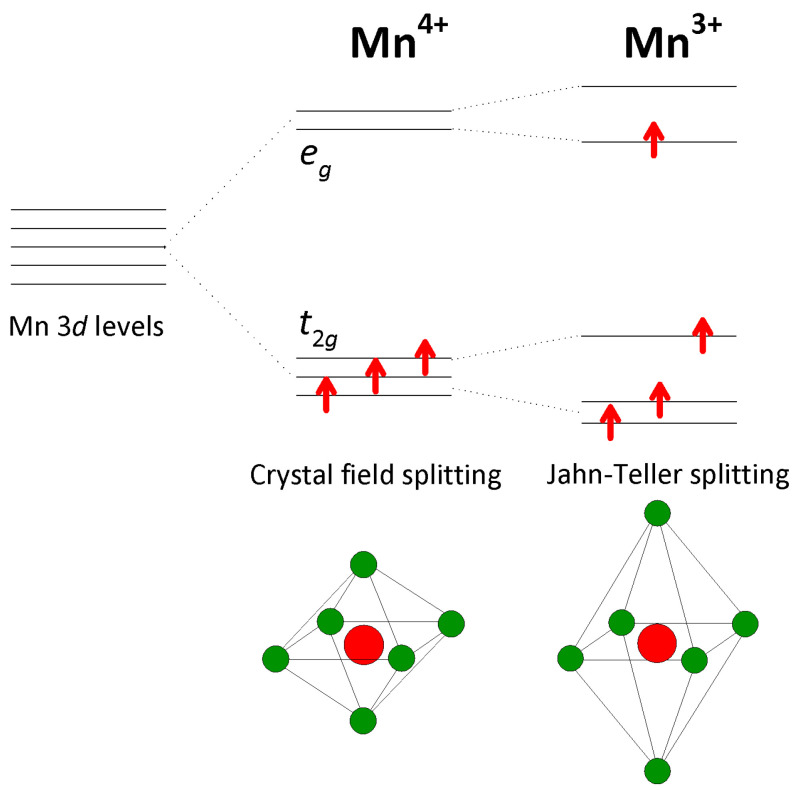
Splitting of the atomic Mn 3*d* levels into high-energy $e_{g}$ and low-energy $t_{2g}$ orbitals in the crystal field created by octahedral surrounding of O atoms. In the case of ${\mathrm{Mn}}^{3+}$, there is additional splitting caused by Jahn–Teller elongation distortion of the ${\mathrm{MnO}}_{6}$ octahedron [5] (see text). Valence electrons are shown as red arrows, Mn atoms as red circles, and O atoms as green circles.

Rich physics of manganites $R_{1-x}A_{x}{\mathrm{MnO}}_{3}$ stems from the mixed valence state ${\mathrm{Mn}}^{3+}{\mathrm{/Mn}}^{4+}$ caused by the substitution of trivalent $R^{3+}$ with a divalent $A^{2+}$. The ${\mathrm{Mn}}^{3+}{\mathrm{/Mn}}^{4+}$ cations are octahedrally surrounded by $O^{2-}$ anions within the distorted perovskite crystal structure, which leads to standard splitting of the atomic Mn 3*d* levels into high-energy $e_{g}$ and low-energy $t_{2g}$ orbitals. In the case of ${\mathrm{Mn}}^{3+}$, there is additional splitting caused by Jahn–Teller elongation distortion of the ${\mathrm{MnO}}_{6}$ octahedron (Figure 1). Three valence electrons of ${\mathrm{Mn}}^{4+}$ occupy the three low-energy $t_{2g}$ orbitals, while four valence electrons of ${\mathrm{Mn}}^{3+}$ occupy the $t_{2g}$ orbitals and the lower of the two $e_{g}$ orbitals. Due to the strong Hund coupling, each orbital is occupied by a single electron, and the spins of all electrons are parallel to each other so that ${\mathrm{Mn}}^{3+}$ and ${\mathrm{Mn}}^{4+}$ have magnetic moments of $4\mu_{B}$ and $3\mu_{B}$, respectively, where $\mu_{B}$ is the Bohr magneton [5].

The strong Hund coupling is the reason why the most relevant hopping process in manganites is the hop of an $e_{g}$ electron from an ${\mathrm{Mn}}^{3+}$ to a neighboring ${\mathrm{Mn}}^{4+}$ cation. It was shown already in 1951 by Zener [6], and later by Anderson and Hasegawa [7] as well as by Kubo and Ohata [8], that the probability *t* of such a hop of the conduction $e_{g}$ electron depends on the angle θ between the magnetic moments of the corresponding ${\mathrm{Mn}}^{3+}$ and ${\mathrm{Mn}}^{4+}$ cations (created by core $t_{2g}$ electrons) t∝cos(θ/2). This is the famous double exchange model that is crucial for understanding of the physics of manganites. According to this model, the maximum hopping probability occurs for the parallel arrangement of neighboring Mn magnetic moments (θ=0), which qualitatively explains why the FM phase in manganites is usually also metallic and why an external magnetic field drastically reduces the resistivity (the CMR effect). Conversely, the minimum (zero) hopping probability occurs for the antiparallel arrangement of neighboring Mn magnetic moments (θ=π), which qualitatively explains why the AFM phase in manganites is also insulating.

Aside from this double exchange interaction that favors the FM arrangement of neighboring Mn magnetic moments, in manganites, there is also the more conventional superexchange interaction between $t_{2g}$ and $e_{g}$ electrons that favors the AFM arrangement of the neighboring Mn magnetic moments [9,10,11,12]. Additionally, manganites are characterized also by a strong electron–phonon interactions, since each hop of a conduction $e_{g}$ electron is accompanied by a structural change in ${\mathrm{MnO}}_{6}$ octahedra induced by the Jahn–Teller effect (see Figure 1), which prefers a polaronic insulating state [13,14,15,16,17,18]. Finally, there are Coulomb interactions among conduction $e_{g}$ electrons, which are believed to be responsible for tendencies towards a charge ordered (CO) insulating state [19,20,21,22,23,24,25].

All these different interactions in manganites are of comparable strength, which is precisely the reason for the aforementioned very rich phase diagrams. Moreover, the competition between different interactions in manganites is so strong that it leads to the so-called nanoscale phase separation, i.e., to the coexistence of nanoclusters of competing phases. The most important is the coexistence of FM and AFM nanoclusters, which is especially pronounced close to the doping *x* that separates the FM metallic and CO/AFM insulating ground states [2,5,26,27,28]. Such a phase-separated state is extremely sensitive to internal tuning parameters such as doping and R/A disorder, as well as to external tuning parameters such as temperature (*T*) and magnetic field. In the case of non-single-crystalline samples, the phase-separated state is also sensitive to the sample’s microstructure, i.e., to grain boundaries in polycrystalline samples [29,30,31,32,33,34,35,36] or to the film/substrate interface in thin film samples [37,38,39,40,41,42,43].

The nanoscale phase separation is believed to be key in understanding the physical properties of manganites, most notably the CMR effect and the transition between the FM metallic and CO/AFM insulating phase [2,5,28,43,44]. One of the most important unresolved problems in manganites is concerned with the conduction mechanism in this FM/AFM phase-separated state. The crucial question here is whether this separated state should be regarded as a mixture of metallic and insulating islands within a random resistor network percolation model, or as a magnetic disorder that additionally scatters the conduction $e_{g}$ electrons. Another important question is concerned with the conduction mechanism in high magnetic fields, when all FM clusters are oriented in the direction of the field. A final important issue is the role of other interactions and the structural disorder in the conduction mechanism of manganites.

In order to address these questions, here, we report our magnetotransport study on sintered nanocrystalline samples of very poorly explored manganites ${\mathrm{Ca}}_{1-x}{\mathrm{Gd}}_{x}{\mathrm{MnO}}_{3}$ in high magnetic fields up to 16 T and at low temperatures down to 2 K. We focus on the samples with low Gd doping levels x=0.05 and x=0.10, because for these samples, previous magnetic measurements indicated a significant phase separation into FM regions within the AFM matrix [45]. Additionally, in these samples, there are no confounding effects related to charge ordering (which occurs at higher *x* values), the AFM phase has the simplest G-type structure, and complications related to the Jahn–Teller distortion of ${\mathrm{Mn}}^{3+}$ are highly reduced [46,47], which makes them an excellent playground for the proposed study of the conduction mechanism in a FM/AFM phase-separated state. In contrast to common beliefs, our results imply that both studied samples x=0.05 and x=0.10 at low temperatures exhibit metallic rather than insulating behavior. We find that this metallic behavior features a peculiar resistivity upturn at the lowest temperatures that can be best fitted to the Kondo-like behavior. The evolution of the fitting parameters with magnetic field points towards a classic electrical percolation picture where the conducting component refers to the FM metallic regions and the non-conducting component refers to the insulating AFM matrix.

## 2. Materials and Methods

Nanopowders of ${\mathrm{Ca}}_{1-x}{\mathrm{Gd}}_{x}{\mathrm{MnO}}_{3}$ with x=0.05 and x=0.10 were prepared using a modified glycine nitrate procedure described in refs. [48,49]. The as-prepared nanopowders were then uniaxially cold pressed at a pressure of 50 MPa and further isostatically pressed at a pressure of 250 MPa. The final sintered nanocrystalline samples in the form of compacts were obtained via thermal pre-etching at temperatures of 1300 and $1400^{\circ }$ for 15 min [45]. Both x=0.05 and x=0.10 sintered samples were reported to have a single phase with a perovskite crystal structure (orthorhombic space group $P_{nma}$, No. 62) and a chemical composition that is in good agreement with the nominal values. The grain size was found to be a few μm, and the crystallite size was found to be around 30 nm.

Resistivity measurements were performed using the standard four-contact dc technique within a commercial Quantum Design Physical Property Measurement System (PPMS) in the temperature range of 2–300 K and in magnetic fields of up to 16 T. Typical sample dimensions for transport measurements were $6\times 3\times 1\;{\mathrm{mm}}^{3}$. The annular electric contacts were prepared by applying silver paste directly to the sample surface. The current was applied along the long axis of the sample, and the magnetic field was applied perpendicular to the current.

## 3. Results

**Figure 2:**
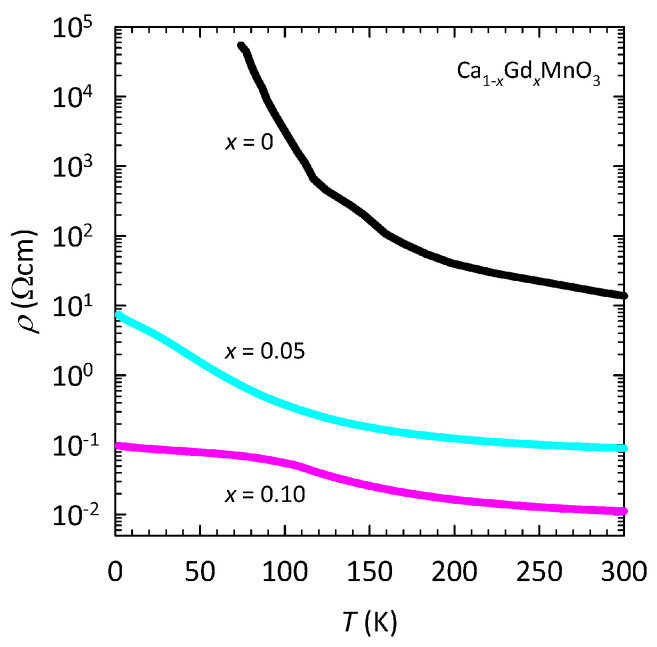
Temperature dependence of resistivity for the ${\mathrm{Ca}}_{1-x}{\mathrm{Gd}}_{x}{\mathrm{MnO}}_{3}$ samples with x=0.05 (cyan line) and x=0.10 (pink line). For comparison, the resistivity curve for the parent compound ${\mathrm{CaMnO}}_{3}$ from ref. [50] is also shown (black line).

The resistivity as a function of temperature for both ${\mathrm{Ca}}_{1-x}{\mathrm{Gd}}_{x}{\mathrm{MnO}}_{3}$ samples x=0.05 and x=0.10 is shown in Figure 2. For comparison, the *T*-dependence of resistivity for the parent compound ${\mathrm{CaMnO}}_{3}$ is also shown [50], which, according to the conventional phase diagram, has an insulating ground state with a single magnetic order, which has the simplest G-type AFM structure [2,3]. As we can see, the Gd doping drastically reduces the resistivity of ${\mathrm{CaMnO}}_{3}$, especially at low *T* values, and more effectively for x=0.10 than for x=0.05. Note that the resistivity derivative dρ/dT for both samples remains <0, which is usually in the literature referred to as insulator-like behavior. However, as we will argue later, both samples with cooling exhibit an insulator-to-metal (IM) crossover, even at H=0, where dρ/dT<0.

**Figure 3:**
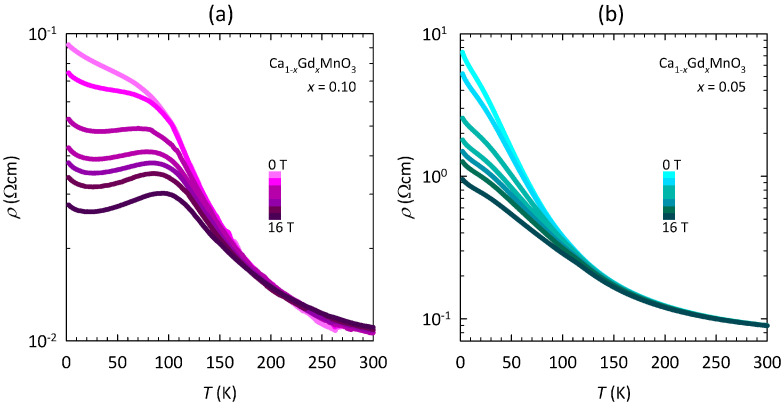
Temperature dependence of resistivity in applied magnetic field for the ${\mathrm{Ca}}_{1-x}{\mathrm{Gd}}_{x}{\mathrm{MnO}}_{3}$ samples with (**a**) x=0.10 and (**b**) x=0.05. Discrete color gradation from light pink (cyan) to dark pink (cyan) corresponds to the increasing field strengths: 0, 1, 5, 8, 10, 12, and 16 T for x=0.10 (x=0.05), respectively.

The effect of an external magnetic field on the resistivity of the ${\mathrm{Ca}}_{1-x}{\mathrm{Gd}}_{x}{\mathrm{MnO}}_{3}$ samples is shown in Figure 3. As we can see, there is a significant decrease in resistivity in the magnetic field at low *T* values, which for x=0.10 in the maximum field of 16 T even leads to real metallic behavior (dρ/dT>0) below ≈100 K. In other words, there is an IM crossover at ≈100 K where ρ(T) reaches a local maximum (Figure 3a). The IM crossover slightly shifts to lower temperatures with decreasing fields and evolves into a knee-like feature at low fields. A similar knee-like feature, although barely visible, is present also for x=0.05 at all fields. Later, we will argue that this knee-like feature is also related to the IM crossover. Note that the metallic behavior in high fields for x=0.10 exhibits a peculiar resistivity upturn at ≈20 K, the temperature at which the ρ(T) curve passes through a local minimum.

**Figure 4:**
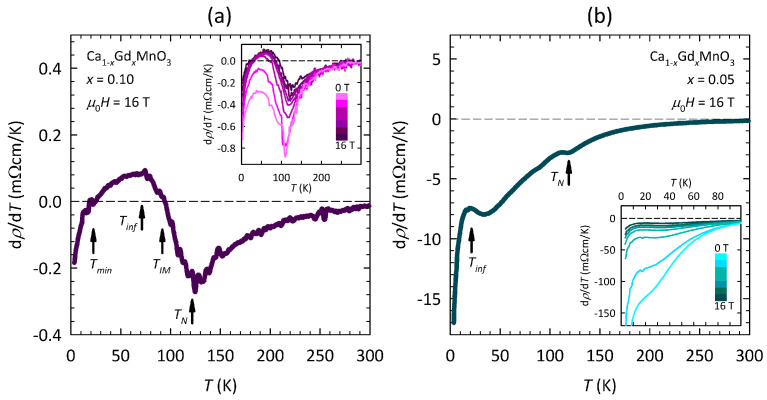
Temperature dependence of resistivity derivative dρ/dT in the maximum magnetic field $\mu_{0}H=16\;T$ for the ${\mathrm{Ca}}_{1-x}{\mathrm{Gd}}_{x}{\mathrm{MnO}}_{3}$ samples with (**a**) x=0.10 and (**b**) x=0.05. Here, $\mu_{0}$ is the vacuum permeability. Characteristic temperatures of $T_{N}$, $T_{IM}$, $T_{inf}$, and $T_{min}$ are indicated by black arrows. The *T*-dependence of dρ/dT at all fields is shown in the insets, where different field strengths of 0, 1, 5, 8, 10, 12, and 16 T correspond to the discrete color gradation from light pink (cyan) to dark pink (cyan) for x=0.10 (x=0.05), respectively.

Let us now focus on the most interesting ρ(T) curve in the maximum field of 16 T for the ${\mathrm{Ca}}_{1-x}{\mathrm{Gd}}_{x}{\mathrm{MnO}}_{3}$ sample x=0.10. The complex *T*-dependence of that curve can be much more easily analyzed by taking the first derivative dρ/dT, which is shown in Figure 4a. As can be seen, there are four characteristic temperatures in dρ/dT. The sharp minimum at $T_{N}\approx 120\;K$ can be readily identified as the Néel temperature, i.e., as the phase transition to the G-type AFM ground state reported in the literature [46,47]. The zero point at $T_{IM}\approx 100\;K$ corresponds to the local resistivity maximum in Figure 3a, i.e., to the IM crossover. The wide maximum at $T_{inf}\approx 70\;K$ corresponds to the inflection point of resistivity, and the zero point at $T_{min}\approx 20\;K$ corresponds to the local resistivity minimum (see Figure 3a), the physical meaning of which will be discussed later.

The evolution of dρ/dT with magnetic field for the x=0.10 sample is shown in the inset of Figure 4a. As can be seen, the dρ/dT curves in the zero field and in the field 1 T are negative across the whole *T*-range, i.e., in contrast to the rest of the curves, they do not have the two zero points associated with $T_{IM}$ and $T_{min}$. On the one hand, such behavior might indicate that the conduction mechanism at low fields is different than the conduction mechanism at high fields. On the other hand, a smooth field evolution of dρ/dT, specifically of the wide maximum at $T_{inf}$, the shape of which does not change with the field, points towards the same conduction mechanism at all fields, which will be further corroborated later.

The minimum at $T_{N}$ and the maximum at $T_{inf}$ are present also in the dρ/dT curve for the ${\mathrm{Ca}}_{1-x}{\mathrm{Gd}}_{x}{\mathrm{MnO}}_{3}$ sample x=0.05 in 16 T (Figure 4b). While $T_{N}$ is the same as for x=0.10, $T_{inf}$ has a significantly lower value ≈20 K. Nevertheless, the similarity with the maximum in dρ/dT for x=0.10 in Figure 4a implies that the conduction mechanism in x=0.05 is qualitatively the same, at least in 16 T. As shown in inset of Figure 4b, the maximum in dρ/dT for x=0.05 becomes less pronounced with decreasing fields, and in the zero field (and 1 T), it evolves into a knee-like feature, which, we will later argue, is related to the IM crossover, i.e., to the same conduction mechanism.

Our detailed analysis shows that the complex behavior of resistivity at low temperatures can be best fitted to the simple Kondo model:

(1) $$\rho \left(T\right)=\rho_{0}+qT^{2}+\rho_{K}\left(T=0\right){\left(\frac{{T_{K}^{\prime }}^{2}}{T^{2}+{T_{K}^{\prime }}^{2}}\right)}^{s},$$

where $\rho_{0}$ is residual resistivity, *q* is the strength of the standard Fermi liquid (FL) term, $\rho_{K}\left(T=0\right)$ is zero-temperature Kondo resistivity, $T_{K}^{\prime }=T_{K}/{\left(2^{1/s}-1\right)}^{1/2}$, $T_{K}$ is the Kondo temperature, and the exponent *s* is taken to be 0.225 [51,52,53,54]. The *T*-dependence of resistivity described by Equation (1) is typical for metallic systems with magnetic impurities which, aside from electron scattering on crystal defects (residual resistivity) and electron–electron scattering (FL term), exhibit additional and very specific electron scattering on magnetic impurities, known as the Kondo effect [55]. Here, for the Kondo contribution in Equation (1), we adopt the empirical relation $\rho_{K}\left(T=0\right){\left[{T_{K}^{\prime }}^{2}/\left(T^{2}+{T_{K}^{\prime }}^{2}\right)\right]}^{s}$ used in several transport studies of various Kondo systems [51,52,53,54]. Here, the Kondo temperature $T_{K}$ is defined as the temperature at which the Kondo resistivity is half relative to its zero-temperature value $\rho_{K}\left(T=0\right)$ [51,55].

**Figure 5:**
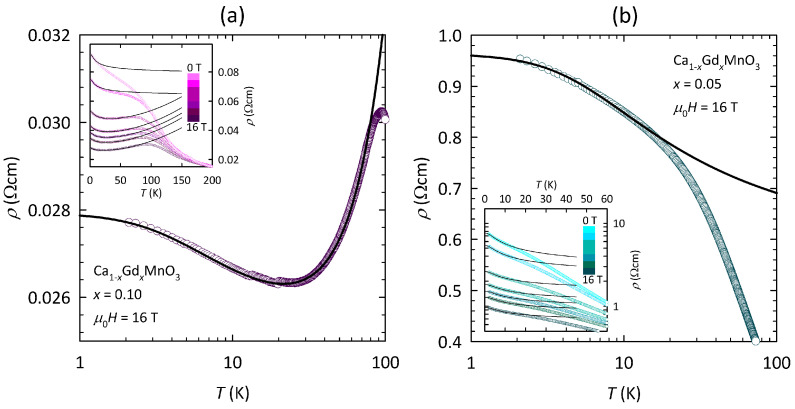
Fitting of the measured resistivity of the ${\mathrm{Ca}}_{1-x}{\mathrm{Gd}}_{x}{\mathrm{MnO}}_{3}$ samples with (**a**) x=0.10 and (**b**) x=0.05 to the simple Kondo model described by Equation (1) in the maximum magnetic field $\mu_{0}H=16\;T$. The fitting procedure at all fields is shown in the insets, where different field strengths of 0, 1, 5, 8, 10, 12, and 16 T correspond to the discrete color gradation from light pink (cyan) to dark pink (cyan) for x=0.10 (x=0.05), respectively. The measured data are shown as empty symbols, and the corresponding fits are given as black lines. To avoid non-physical behavior, the fits of ρ(T) for x=0.10 at 0 and 1 T, as well as the fits of ρ(T) for x=0.05 at all fields, were performed in such a way that the parameter *q* in Equation (1) was forced to be zero (see text).

Fitting of the measured resistivity of the ${\mathrm{Ca}}_{1-x}{\mathrm{Gd}}_{x}{\mathrm{MnO}}_{3}$ samples to the Kondo model described by Equation (1) is shown in Figure 5. Let us first focus on the most instructive fitting of the ρ(T) curve for x=0.10 in the maximum field of 16 T (Figure 5a). As we can see, the logarithmic *T*-scale reveals another feature of the resistivity curve, namely a tendency towards saturation at the lowest *T* value (not visible on the linear *T*-scale in Figure 3a). Furthermore, we can see that the Kondo model covers this feature as well as the local resistivity minimum around ≈20 K and successfully fits the measured resistivity up to ≈80 K, beyond which there are deviations from the Kondo model due to the proximity of the IM crossover. Here, it is worth pointing out that the increase of resistivity below $T_{min}\approx 20\;K$, where dρ/dT<0 (see Figure 4a), does not imply insulator-like behavior but rather metallic behavior with a Kondo-like temperature dependence.

As shown in the inset of Figure 5a, the Kondo model successfully fits the ρ(T) curves of the x=0.10 sample at all applied fields down to 5 T. Here, note that the *T*-interval, in which the fits apply, shrinks with decreasing fields and for 5 T covers the range up to only ≈60 K due to the proximity of the IM crossover. The fitting of the resistivity curves at 0 and 1 T to Equation (1) gives a negative value for the FL parameter *q*, which is not physical. This negative value of *q* is due to the fact that the resistivity for x=0.10 at 0 and 1 T does not follow the true metallic behavior (dρ/dT>0) in any *T*-range (see inset of Figure 4a). Nevertheless, if we constrain the fitting procedure in such a way that the parameter *q* is forced to be zero, it turns out that the Kondo model can reasonably fit even the ρ(T) curves in 0 and 1 T, though in a very limited *T*-range up to only ≈15 K (see inset of Figure 5a). Such behavior implies that the conduction mechanism at low fields is still metallic, but due to the limited *T*-range, only the contribution of Kondo scattering (dρ/dT<0) can be clearly discerned. The FL $T^{2}$ term, which is expected to appear at higher *T* values, is likely masked by the proximity of the IM crossover. At 0 and 1 T, this crossover can be associated with the knee-like feature in ρ(T) in Figure 3a or equivalently with the wide maximum in dρ/dT in the inset of Figure 4a.

Let us now focus on the fitting of the measured resistivity for the ${\mathrm{Ca}}_{1-x}{\mathrm{Gd}}_{x}{\mathrm{MnO}}_{3}$ sample with x=0.05 in 16 T (Figure 5b). For the same reason as for x=0.10 at low fields, here, the fitting procedure was constrained in such a way that the parameter *q* was forced to be zero. As can be seen, such constrained Kondo fitting works reasonably well at low *T* values up to ≈15 K, beyond which there are deviations, likely related to the proximity of the IM crossover, which by analogy with x=0.10 may be associated with the wide maximum in dρ/dT at $T_{inf}$ in Figure 4b. The same is true for ρ(T) curves of x=0.05 for all fields down to 0 T, as indicated by the inset of Figure 5b. Here, the *T*-interval, in which the fits apply, shrinks very little with decreasing fields due to the IM crossover that barely shifts with decreasing field and for 0 T is plausibly associated with the knee-like feature in dρ/dT at ≈20 K (see inset of Figure 4b).

A further confirmation of the viability of the Kondo model can be found in the obtained fitting parameters, which are for both ${\mathrm{Ca}}_{1-x}{\mathrm{Gd}}_{x}{\mathrm{MnO}}_{3}$ samples listed in Table 1. The Kondo temperature $T_{K}$ for both samples and for all fields turns out to be around 20 K, in very good agreement with the typical values in the literature, which span the range of 1–100 K [56] and are commonly around 10–20 K [55]. More importantly, the extracted Kondo temperature agrees very well with those obtained in other rare-earth-doped manganites, such as ${\mathrm{Nd}}_{0.67}{\mathrm{Sr}}_{0.33}{\mathrm{MnO}}_{3}$ [57] and ${\mathrm{Pr}}_{0.67-x}{\mathrm{Bi}}_{x}{\mathrm{Ba}}_{0.33}{\mathrm{MnO}}_{3}$ [58], where $T_{K}$ was found to be in the range of 25–30 K. Furthermore, the obtained residual resistivity $\rho_{0}$ and the zero-temperature Kondo resistivity $\rho_{K}\left(T=0\right)$ for both samples exhibit a monotonous change with magnetic field, which corroborates that the nature of the conduction mechanism does not change going from high to low fields. Why $\rho_{0}$ and $\rho_{K}\left(T=0\right)$ significantly change with magnetic field, while $T_{K}$ and *q* are nearly constant with field, will be discussed in the next section.

## 4. Discussion

The results of our magnetotransport study on sintered nanocrystalline samples of ${\mathrm{Ca}}_{1-x}{\mathrm{Gd}}_{x}{\mathrm{MnO}}_{3}$ with x=0.05 and x=0.10, described in detail in the previous section, clearly show that Gd doping progressively enhances the electric conductivity compared to the parent compound ${\mathrm{CaMnO}}_{3}$, which is a well-known G-type AFM insulator [2,3] (Figure 2). Similar behavior has also been found by other groups for both polycrystalline [46] as well as thin film [47] ${\mathrm{Ca}}_{1-x}{\mathrm{Gd}}_{x}{\mathrm{MnO}}_{3}$ samples. The obtained decrease in resistivity is easy to understand because the substitution of ${\mathrm{Ca}}^{2+}$ with ${\mathrm{Gd}}^{3+}$ introduces the mixed valence state ${\mathrm{Mn}}^{4+}{\mathrm{/Mn}}^{3+}$, which leads to increasing numbers of conduction $e_{g}$ electrons (Figure 1). Based on the standard double exchange model, the hopping of these $e_{g}$ electrons favors the parallel arrangement of Mn magnetic moments, which also leads to an increase in the FM interaction in the system. Indeed, our previous magnetic study on the very same ${\mathrm{Ca}}_{1-x}{\mathrm{Gd}}_{x}{\mathrm{MnO}}_{3}$ samples x=0.05 and x=0.10 indicated the presence of FM clusters in the G-type AFM matrix [45], with a higher fraction in x=0.10, in line with the widely accepted scenario of phase separation in manganites [2,5,26,27,28].

The external magnetic field is shown to have a significant impact on the conducting properties of the studied ${\mathrm{Ca}}_{1-x}{\mathrm{Gd}}_{x}{\mathrm{MnO}}_{3}$ samples, leading to a progressive decrease of resistivity in the field, i.e., to a large negative magnetoresistance (Figure 3). Similar negative magnetoresistance has also been previously found in other polycrystalline [46] and thin film [47] ${\mathrm{Ca}}_{1-x}{\mathrm{Gd}}_{x}{\mathrm{MnO}}_{3}$ samples at low *x* values. Such behavior is not surprising in manganites, especially in the presence of the FM clusters in the AFM matrix, and is usually ascribed to easy alignment and growth of these FM clusters which intensifies the hopping of conduction $e_{g}$ electrons, in turn leading to a significant decrease in resistivity. The same mechanism is at play also in the famous CMR effect, although there are additional effects that need to be considered [43].

The effect of the field on resistivity is shown to be strong only at low temperatures and weakens significantly with heating, becoming negligible at high temperatures (Figure 3). Such behavior implies that the FM clusters appear only at lower temperatures (below $\approx T_{N}$) and that their fraction increases with further cooling, in line with previous magnetic measurements for these samples [45]. Based on the metallic behavior found at low *T* values, which is especially pronounced for the sample x=0.10 at high fields (Figure 3a), it seams plausible that the FM fraction at low *T* values becomes high enough for some of the FM clusters to bind together and form a percolative metallic path through the sample. Such a percolative nature of the metallic state has been proposed for various manganite systems: ${\mathrm{Pr}}_{0.63}{\mathrm{Ca}}_{0.37}{\mathrm{MnO}}_{3}$ [59], ${\mathrm{La}}_{0.33}{\mathrm{Pr}}_{0.34}{\mathrm{Ca}}_{0.33}{\mathrm{MnO}}_{3}$ [60], ${\mathrm{La}}_{0.9}{\mathrm{Te}}_{0.1}{\mathrm{MnO}}_{3}$ [61], ${\mathrm{La}}_{0.5}{\mathrm{Ca}}_{0.5}{\mathrm{Mn}}_{1-x}{\mathrm{Al}}_{x}O_{3-\delta }$ [62], and ${\mathrm{La}}_{1-x}{\mathrm{Ca}}_{x}{\mathrm{MnO}}_{3}$ [36,43,63], implying a more general feature of the electric conduction in manganites.

Within the percolation picture of manganites, the metallic conduction appears only below a certain characteristic temperature at which the FM (metallic) fraction reaches the percolation threshold. In our case, the percolation threshold can be readily associated with the IM crossover, which is most obvious for the ${\mathrm{Ca}}_{1-x}{\mathrm{Gd}}_{x}{\mathrm{MnO}}_{3}$ sample x=0.10 at fields ≥5T, where ρ(T) passes through a local maximum (Figure 3a). For the x=0.10 sample at fields <5T, as well as for the x=0.05 sample at all fields, where ρ(T) does not pass through a local maximum, the percolation threshold might be associated with the maximum or the knee-like feature in dρ/dT (Figure 4).

The most important finding of this study is that the metallic conduction for x=0.05 and x=0.10 beyond the percolation threshold exhibits a peculiar resistivity upturn at the lowest *T* value, which can be described by the simple Kondo model (Figure 5). Namely, the Kondo effect is a very specific scattering mechanism, typical for metallic systems with magnetic impurities, resulting from a virtual exchange of spin state between the conduction electron and magnetic impurity [55,56]. Kondo scattering has been rarely considered in manganites because most of the studies on manganites $R_{1-x}A_{x}{\mathrm{MnO}}_{3}$ have been conducted on compounds in which the randomly distributed rare-earth cation $R^{3+}$ is non-magnetic ${\mathrm{La}}^{3+}$ [2,3,4,5,64]. In our study, however, the randomly distributed rare-earth cation is ${\mathrm{Gd}}^{3+}$, which has a large magnetic moment $7\mu_{B}$. The Kondo-like behavior in our ${\mathrm{Ca}}_{1-x}{\mathrm{Gd}}_{x}{\mathrm{MnO}}_{3}$ samples might therefore be ascribed precisely to the scattering resulting from an exchange of spin between the conduction electron and magnetic ${\mathrm{Gd}}^{3+}$ cation. A direct confirmation of this mechanism could be provided via specific heat or magnetic susceptibility measurements, which is left for a future study.

Here, it is worth mentioning that Kondo scattering is usually found in systems with magnetic impurities, the magnetic moment of which is much smaller than that of ${\mathrm{Gd}}^{3+}$. Especially interesting are studies that associate Kondo scattering with the peculiar resistivity upturn at low *T* values in less common manganites: (${\mathrm{La}}_{0.1}{\mathrm{Ce}}_{0.4}{\mathrm{Sr}}_{0.5}$)${\mathrm{MnO}}_{3}$ [65], ${\mathrm{Nd}}_{0.67}{\mathrm{Sr}}_{0.33}{\mathrm{MnO}}_{3}$ [57], ${\mathrm{Pr}}_{0.67-x}{\mathrm{Bi}}_{x}{\mathrm{Ba}}_{0.33}{\mathrm{MnO}}_{3}$ [58], ${\mathrm{La}}_{0.75}{\mathrm{Sr}}_{0.20}{\mathrm{Mn}}_{1-x}{\mathrm{Co}}_{x}O_{3}$ [66], and ${\mathrm{La}}_{1-x}{\mathrm{Hf}}_{x}{\mathrm{MnO}}_{3}$ [67]. In this regard, the Kondo scattering in the system containing ${\mathrm{Gd}}^{3+}$ with a large magnetic moment is somewhat surprising. According to the theory, however, the magnitude of the impurity’s magnetic moment is not crucial for the Kondo effect. Instead, its coupling to the conduction electrons is important, which must be antiferromagnetic [55]. Moreover, according to the theory, the Kondo contribution to the resistivity increases with the impurity’s magnetic moment, implying that the systems with ${\mathrm{Gd}}^{3+}$ might exhibit even stronger Kondo scattering than systems with other rare-earth elements which have smaller moments, of course, assuming similar antiferromagnetic coupling strength.

In all of the aforementioned previous studies [57,58,65,66,67], the Kondo contribution has been modeled by a conventional theoretically predicted Kondo relation that implies a logarithmic *T*-dependence ρ(T)∝−lnT [55,56]. Our study, however, clearly shows that the resistivity of both studied ${\mathrm{Ca}}_{1-x}{\mathrm{Gd}}_{x}{\mathrm{MnO}}_{3}$ samples x=0.05 and x=0.10 strongly deviates from such a logarithmic *T*-dependence and instead tends towards saturation at the lowest *T* value (Figure 5). As shown in the previous section, this Kondo contribution can be modeled only by using the empirical relation for the Kondo term $\rho \left(T\right)=\rho_{K}\left(T=0\right){\left[{T_{K}^{\prime }}^{2}/\left(T^{2}+{T_{K}^{\prime }}^{2}\right)\right]}^{s}$ in Equation (1), put forward by several studies of various Kondo systems [51,52,53,54]. This empirical relation removes the non-physical singular behavior of the lnT term at zero-temperature and accurately predicts saturation of the Kondo contribution towards T=0, thus better reflecting the behavior of real systems [55,68]. The exponent *s* in the empirical relation is usually taken to be 0.225 in order to closely match the theoretical result obtained by the numerical renormalization group [51,52]. Although this numerical prediction was calculated for an impurity with the magnetic moment $1\mu_{B}$, here, we show that the same result can also reasonably fit our data for the Gd impurity with the magnetic moment $7\mu_{B}$. It is worth mentioning that we have considered alternative quantum correction models as well, such as weak-localization, Coulomb interactions, and variable-range hopping [54,58,66,67]. However, none of these have been able to capture the obtained resistivity upturn, especially its saturation as T→0.

In contrast to the aforementioned less common manganites [53,54,57,58,66,67], our study shows that the metallic behavior in ${\mathrm{Ca}}_{1-x}{\mathrm{Gd}}_{x}{\mathrm{MnO}}_{3}$ can be modeled by a simple expression that, aside from the Kondo term, contains only the additional conventional FL term $\rho_{0}+qT^{2}$ (Equation (1)). We further show that some of the fitting parameters exhibit a pronounced dependence on external magnetic field (Table 1), which is not surprising given the obtained large negative magnetoresistance (Figure 3). Nevertheless, the field dependence of the fitting parameters deserves closer attention.

Let us now focus on the residual resistivity $\rho_{0}$, which describes a resistivity contribution that comes from elastic scattering of conduction electrons on crystal defects. First of all, it is worth mentioning that the residual resistivity of our ${\mathrm{Ca}}_{1-x}{\mathrm{Gd}}_{x}{\mathrm{MnO}}_{3}$ samples with x=0.05 and x=0.10 is significantly larger than the residual resistivity of the corresponding samples in the study by Maignan et al. [69]. Such behavior might be related to the presence of very small crystallites in our samples of only 30 nm, which gives rise to additional elastic scattering of conduction electrons on crystallite boundaries. Furthermore, as can be seen in Table 1, $\rho_{0}$ for both ${\mathrm{Ca}}_{1-x}{\mathrm{Gd}}_{x}{\mathrm{MnO}}_{3}$ samples was found to strongly decrease with the magnetic field. Since the scattering on crystal defects is not expected to change in a magnetic field, the only plausible explanation for the obtained decrease in $\rho_{0}$ in the field can be offered by applying the classical percolation picture in the regime just beyond the electrical percolation threshold. According to such percolation picture:

(2) $$\rho =\rho_{f}{\left(V_{f}-V_{c}\right)}^{-p},$$

where ρ is the electrical resistivity of the composite (mixture of a conducting and a non-conducting component), $\rho_{f}$ is the electrical resistivity of the filler (the conducting component), $V_{f}$ is the filler volume fraction, $V_{c}$ is the filler volume fraction at the percolation threshold, and *p* is the critical percolation exponent, which for a 3D filler network lies between 1.6 and 2 [70,71,72,73,74,75,76]. In the case of our ${\mathrm{Ca}}_{1-x}{\mathrm{Gd}}_{x}{\mathrm{MnO}}_{3}$ samples, the composite refers to the phase-separated state of FM metallic regions within an insulating AFM matrix, and the filler refers to the FM metallic regions. Within this percolation picture, the obtained negative magnetoresistance, i.e., the decrease in measured resistivity in the magnetic field (Figure 3), should be attributed entirely to the field-induced increase in the FM metallic fraction $V_{f}$ without any changes in the intrinsic resistivity of the FM metallic component $\rho_{f}$. A similar consideration also applies to the obtained decrease in residual resistivity $\rho_{0}$ in the magnetic field (Table 1), which should be attributed to the growth of the FM metallic fraction $V_{f}$ rather than to changes in the intrinsic scattering mechanism.

The same percolation model can also nicely explain the field dependence of the Kondo-like contribution in both ${\mathrm{Ca}}_{1-x}{\mathrm{Gd}}_{x}{\mathrm{MnO}}_{3}$ samples, specifically why the obtained zero-temperature Kondo resistivity $\rho_{K}\left(T=0\right)$ strongly decreases with magnetic field while the corresponding Kondo temperature $T_{K}$ stays constant (Table 1). Namely, by comparing Equations (1) and (2), it becomes apparent that in the Kondo term $\rho_{K}\left(T=0\right){\left[{T_{K}^{\prime }}^{2}/\left(T^{2}+{T_{K}^{\prime }}^{2}\right)\right]}^{s}$, only $\rho_{K}\left(T=0\right)$ should be affected by the field-induced increase in the FM metallic fraction $V_{f}$, while $T_{K}^{\prime }=T_{K}/{\left(2^{1/s}-1\right)}^{1/2}$ and consequently $T_{K}$ should stay unchanged. The insensitivity of $T_{K}$ to the field has a physical explanation as well, since within the Kondo model, $T_{K}$ depends on the energy and the width of the impurity’s atomic level, as well as on the Coulomb repulsion energy between two electrons at the site of the impurity [56], which are all atomic quantities that are not expected to significantly change in magnetic field.

Finally, let us focus on the last fitting parameter, the FL parameter *q*, which is related to inelastic electron–electron scattering and was successfully extracted only for the ${\mathrm{Ca}}_{1-x}{\mathrm{Gd}}_{x}{\mathrm{MnO}}_{3}x=0.10$ sample and only for fields ≥5T (Table 1). According to the percolation model, the parameter *q* should follow the same evolution with magnetic field as the parameters $\rho_{0}$ and $\rho_{K}\left(T=0\right)$, which follows naturally from the comparison between Equations (1) and (2). However, despite the limited field range, it can be clearly seen that *q* does not change with field, in contradiction to the proposed percolation scenario.

The percolation scenario in our ${\mathrm{Ca}}_{1-x}{\mathrm{Gd}}_{x}{\mathrm{MnO}}_{3}$ samples with x=0.05 and x=0.10 has at least two additional challenges. The first one is related to the FM metallic fraction $V_{f}$, which at least in high magnetic fields is expected to be proportional to the magnetization of the sample [36,43]. Based on the obtained field evolution of the fitting parameters $\rho_{0}$ and $\rho_{K}\left(T=0\right)$ (Table 1), our magnetotransport study implies that the FM metallic fraction $V_{f}$ for both ${\mathrm{Ca}}_{1-x}{\mathrm{Gd}}_{x}{\mathrm{MnO}}_{3}$ samples continues to significantly increase even at the highest fields of up to 16 T. However, our previous magnetic study conducted on the very same ${\mathrm{Ca}}_{1-x}{\mathrm{Gd}}_{x}{\mathrm{MnO}}_{3}$ samples indicates that the magnetization and therefore $V_{f}$ tends towards saturation already in fields up to only 5T [45]. The second challenge is related to the quantitative change in the fitting parameters $\rho_{0}$ and $\rho_{K}\left(T=0\right)$, which should within the percolation scenario both change with field by the same factor determined solely by $V_{f}$. However, our study clearly implies that the zero-temperature Kondo resistivity $\rho_{K}\left(T=0\right)$ changes faster with field than the residual resistivity $\rho_{0}$, which is especially pronounced for the sample x=0.05 (Table 1).

These deviations from the percolation scenario could point towards the presence of additional contributions to the obtained negative magnetoresistance in our ${\mathrm{Ca}}_{1-x}{\mathrm{Gd}}_{x}{\mathrm{MnO}}_{3}$ samples. One contribution might be associated with the Kondo effect itself, as several studies have reported negative magnetoresistance related solely to Kondo scattering in a magnetic field [53,54,77,78,79]. Another contribution might be associated with a magnetic structure that is more complex than the proposed G-type AFM matrix with FM islands, due to which the relation between the FM metallic fraction and the magnetization would not be as trivial as the one assumed here. Finally, there might be a contribution from tunnel magnetoresistance effect across boundaries between FM metallic and AFM insulating regions, considered in some previous studies of manganites [80,81]. In order to resolve these issues, further experimental and theoretical studies are needed, especially ones based on magnetic measurements at high fields and low temperatures, preferably with spatial resolution.

## 5. Conclusions

Our magnetotransport study on sintered nanocrystalline ${\mathrm{Ca}}_{1-x}{\mathrm{Gd}}_{x}{\mathrm{MnO}}_{3}$ samples shows that light Gd doping x=0.05 and x=0.10 not only drastically reduces the resistivity of the parent antiferromagnetic insulator ${\mathrm{CaMnO}}_{3}$ but even leads to metallic conduction at low temperatures. This metallic conduction is found to exhibit a peculiar resistivity upturn at the lowest temperatures, which can be well fitted by the simple Kondo model and possibly be ascribed to Kondo-like scattering of conduction electrons on Gd impurities. We also show a large negative magnetoresistance that can be most easily understood within a classical electrical percolation picture where the conducting component refers to ferromagnetic metallic islands and the non-conducting component refers to the antiferromagnetic insulating matrix. The complex evolution of the metallic conduction in a magnetic field, however, points towards the presence of additional contributions to the negative magnetoresistance that might be related to the field-induced suppression of Kondo scattering and/or tunnel magnetoreistance effect. The identification of these additional contributions and their relation to the percolation picture requires further experimental and theoretical studies.

## Figures and Tables

**Table 1:** Extracted parameters from the fitting of the measured resistivity of the ${\mathrm{Ca}}_{1-x}{\mathrm{Gd}}_{x}{\mathrm{MnO}}_{3}$ samples with x=0.10 and x=0.05 to the Kondo model described by Equation (1) and shown in Figure 5. The error bars of the fitting parameters $\rho_{0}$, *q*, and $\rho_{K}\left(T=0\right)$ reflect 20% uncertainty in sample geometry, and the fitting parameter $T_{K}$ reflects the uncertainty in the fitting procedure.

		x=0.10				x=0.05		
$\mu_{0}H$ (T)	$\rho_{0}$ ($10^{-2}\;\Omega$cm)	*q* ($10^{-7}\;\Omega \mathrm{cm}K^{-2}$)	$\rho_{K}\left(T=0\right)$ ($10^{-3}\;\Omega$cm)	$T_{K}$ (K)	$\rho_{0}$ (Ωcm)	*q* ($10^{-7}\;\Omega \mathrm{cm}K^{-2}$)	$\rho_{K}\left(T=0\right)$ (Ωcm)	$T_{K}$ (K)
0	7.8±1.5	-	14.8±3.0	20±7	2.0±0.4	-	5.6±1.1	19±7
1	6.3±1.3	-	12.3±2.5	19±7	1.9±0.4	-	3.5±0.7	19±7
5	4.3±0.9	7.9±1.6	10.0±2.0	21±5	1.3±0.3	-	1.3±0.3	19±7
8	3.5±0.7	8.3±1.7	7.7±1.5	18±5	1.0±0.2	-	0.8±0.2	22±7
10	3.2±0.6	8.3±1.7	6.5±1.3	19±5	0.9±0.2	-	0.7±0.1	22±7
12	2.9±0.6	8.3±1.7	5.6±1.1	20±5	0.8±0.2	-	0.5±0.1	21±7
16	2.4±0.5	7.4±1.5	3.5±0.7	16±5	0.6±0.1	-	0.4±0.1	22±7

## Data Availability

The data are contained within the article.
